# Maternal anxiety during pregnancy is associated with weaker prefrontal functional connectivity in adult offspring

**DOI:** 10.1007/s11682-023-00787-1

**Published:** 2023-06-29

**Authors:** Elise Turk, Marion I. van den Heuvel, Charlotte Sleurs, Thibo Billiet, Anne Uyttebroeck, Stefan Sunaert, Maarten Mennes, Bea R.H. Van den Bergh

**Affiliations:** 1 Department of Cognitive Neuropsychology, Tilburg University, Warandelaan 2, 5037AB Tilburg, The Netherlands; 2 Department of Oncology, Catholic University of Leuven, KU Leuven, Leuven, Belgium; 3 Icometrix, Leuven, Belgium; 4 Radiology, KU Leuven, Leuven, Belgium; 5 Donders Institute for Brain, Cognition and Behaviour, Radboud University Nijmegen, Nijmegen, The Netherlands; 6 Health Psychology Research Group, Catholic University of Leuven, KU Leuven, Leuven, Belgium; 7 Department of Welfare, Public Health and Family, Flemish Government, Brussels, Belgium

**Keywords:** Fetal programming, Resting-state functional connectivity, Prospective study, Medial prefrontal cortex, Prenatal stress

## Abstract

**Background:**

The connectome, constituting a unique fingerprint of a person’s brain, may be influenced by its prenatal environment, potentially affecting later-life resilience and mental health.

**Methods:**

We conducted a prospective resting-state functional Magnetic Resonance Imaging study in 28-year-old offspring (N = 49) of mothers whose anxiety was monitored during pregnancy. Two offspring anxiety subgroups were defined: “High anxiety” (n = 13) group versus “low-to-medium anxiety” (n = 36) group, based on maternal self-reported state anxiety at 12–22 weeks of gestation. To predict resting-state functional connectivity of 32 by 32 ROIs, maternal state anxiety during pregnancy was included as a predictor in general linear models for both ROI-to-ROI and graph theoretical metrics. Sex, birth weight and postnatal anxiety were included as covariates.

**Results:**

Higher maternal anxiety was associated with weaker functional connectivity of medial prefrontal cortex with left inferior frontal gyrus (*t* = 3.45, *p*_*FDR*_ < 0.05). Moreover, network-based statistics (NBS) confirmed our finding and revealed an additional association of weaker connectivity between left lateral prefontal cortex with left somatosensory motor gyrus in the offspring. While our results showed a general pattern of lower functional connectivity in adults prenatally exposed to maternal anxiety, we did not observe significant differences in global brain networks between groups.

**Conclusions:**

Weaker (medial) prefrontal cortex functional connectivity in the high anxiety adult offspring group suggests a long-term negative impact of prenatal exposure to high maternal anxiety, extending into adulthood. To prevent mental health problems at population level, universal primary prevention strategies should aim at lowering maternal anxiety during pregnancy.

**Supplementary Information:**

The online version contains supplementary material available at 10.1007/s11682-023-00787-1.

## Introduction

Worldwide the societal burden of mental health problems is increasing (Vigo et al., 2016). Although early stage prevention is more cost-efficient than treatment (Bauer et al., 2016), prenatal origins of mental health problems that are often preventable remain understudied (Browne et al., 2020; Glover, 2011; Monk et al., 2019; van den Heuvel, 2022). In a UK-based study, it has been estimated that perinatal anxiety and depression combined costs the society about £8500 per woman giving birth. This results in a striking £6.6 billion in total costs (for both mother and child) for the United Kingdom alone (Bauer et al., 2016). The majority of these costs were associated with adverse effects of maternal perinatal depression on the children, emphasizing the need for more research on the underlying mechanisms of adverse consequences of maternal psychological distress during pregnancy, especially on the long-term.

More than a decade of brain imaging research has shown that maternal psychological distress during pregnancy, including depression and anxiety, affects the developing fetal brain, with later life consequences for offspring’s cognition and mental health (for a review, see Adamson et al., 2018; Dufford et al., 2021; Pulli et al., 2019; Van den Bergh et al., 2018). Recent studies found evidence for changes in offspring’s structural grey matter (e.g., Acosta et al., 2019, 2020; Donnici et al., 2021; Moog et al., 2021) and white matter (e.g., Demers et al., 2021; Manning et al., 2022; Rifkin-Graboi et al., 2015) as well as functional brain changes, including resting-state functional connectivity (rsFC) using fMRI (e.g., Humphreys et al., 2020; Manning et al., 2022; Rajasilta et al., 2023; Scheinost et al., 2020) and task-based fMRI studies (e.g., Mennes et al., 2020; van der Knaap et al., 2018). Several pioneering studies have even started to show that the timing of these brain alterations is prenatally, by studying the offspring *in utero* with fetal resting-state fMRI (De Asis-Cruz et al., 2020; Thomason et al., 2021; van den Heuvel et al., 2021; Wu et al., 2022). Such neural alterations potentially underlie the observed behavioral problems and mental health issues of prenatally exposed offspring (Monk et al., 2019; Van den Bergh et al., 2018).

While studies on developmental origins of infant and child brain development are still increasing, only very few studies examined the lasting effect of prenatal exposure to maternal distress into puberty or adulthood. Consequently, we lack knowledge about the persistence of brain developmental alterations in the aftermath of prenatal exposure to maternal distress. Prospective pregnancy cohorts that continue into adulthood may add incredibly valuable information, especially since several researchers have pointed out that neurodegenerative disorders, such as Parkinson’s and Altzheimer’s Disease, may find their origin in fetal life (Boots et al., 2023; Faa et al., 2014). The limited number of studies that do exist clearly show persistent brain alteration into adulthood, such as presumed accelerated brain aging in young adults prenatally exposed to maternal depression (Mareckova et al., 2020), and a deficit in endogenous cognitive control in 20-year-old males as measured with task-based fMRI (Mennes et al., 2020). Still, more prospective research with longer follow-up periods are necessary.

Additionally, an important gap in neuroimaging research to date is the focus on predetermined brain areas. Most research has focused on structural changes of the amygdala and hippocampus or rsFC of the limbic and/or (pre)frontal region (Scheinost et al., 2017). Even though several studies have found important results in changing brain structure and function of these brain regions (Acosta et al., 2019, 2020; Donnici et al., 2021; Humphreys et al., 2020; Jones et al., 2019; Scheinost et al., 2016, 2020; van der Knaap et al., 2018), this targeted approach may miss important changes to global brain function and network properties of the prenatally exposed brain (Scheinost et al., 2017). Exploration of the adult whole brain network prenatally exposed to maternal distress, with appropriate control for multiple testing, has not been conducted to date.

In the current study, we utilize a unique prospective prenatal cohort with a postnatal follow-up of 28-years to study the long-term effects of prenatal exposure to maternal anxiety on whole brain functional connectivity. To this aim, we gathered rs-fMRI scans of the adult offspring to evaluate its association with maternal anxiety at 12–22 weeks of pregnancy. We examined resting-state functional connectivity both locally, by studying differences in ROI-to-ROI connectivity using 32 cortical and cerebral ROIs, and by studying the whole-brain network properties with graph metrics, using two different atlases. We expected to observe altered brain functional connectivity associated with prenatal exposure to maternal anxiety. Given the lack of adult offspring research, a data-driven approach was implemented, with no a priori expectations on directions of effects.

## Methods

### Study design

From the 86 pregnant women that initially participated in 1986 in a prospective longitudinal study, 52 of their offspring participated in our study at age 28 years. Inclusion criteria at the start of the study were Caucasian race, Dutch speaking, aged between 18 and 30 weeks pregnant, nulliparous and without obstetrical complications or medical risks, and not using drugs or medication with risks to the fetus (Van den Bergh, 1990; Van den Bergh & Marcoen, 2004). None of the mothers used SSRI or glucocorticoids and none used drugs other than alcohol, nicotine products and prescription drugs used for medical purposes. Maternal data assessment included the State Trait Anxiety Inventory which was used to screen for anxiety symptoms during weeks 12–22 and was also taken at, 23–31 and 32–40 weeks of pregnancy and at several waves postnatally.

The 28-year-old offspring participated in this study between July 2014 and September 2015 in a University Hospital. The rsFC analyses were performed on the available high-quality imaging data of 49 subjects (after exclusion of one case of whom the T1 image was not available and two cases with Root Mean Square (RMS) motion parameters > 1 mm in rs-fMRI images; N = 3). Demographic characteristics of the total group of mothers and offspring (N = 49, final sample) are presented in Table 1 and **Supplemental demographics, Table** S1. As recommended by Pulli et al. (2019), we reported on offspring age at MRI scan, gestational age at birth, offspring sex, birth weight, maternal age, maternal BMI, race/ethnicity, socioeconomic status, and drug, alcohol, and tobacco use during pregnancy.

**Table 1:** Demographics and descriptive statistics between Low-Medium Anxiety group and High Anxiety group

Variables	Follow-up sample mean (SD)	Low-Medium anxiety (LMA) group mean (SD)	High-anxiety (HA) group mean (SD)	Independent sample t-test*
Parents	(n = 48)	(n = 35)	(n = 13)	
Maternal state anxiety 12–22 weeks of pregnancy	38.84 (8.74)	34.59 (4.73)	50.29 (6.44)	**-9.24 (*p*** **< .001)**
Postnatal maternal trait anxiety	0.12 (0.99)	− 0.16 (0.95)	0.89 (0.61)	**-3.7 (*p*** **< .001)**
Maternal age at 12–22 weeks, years	26.19 (2.55)	25.89 (2.54)	27 (2.48)	-1.36 (*p* = .18)
Paternal age at 12–22 weeks, years	28.32 (4.16)	28.16 (4.44)	28.75 (3.44)	174.5 (*p* = .65)
Social class (based on education both parents)	0.20 (0.99)	0.3 (0.95)	− 0.07 (1.09)	280.50 (*p* = .21)
Months married	36.5 (25.67)	38.03 (25.98)	31.64 (25.21)	232 (*p* = .31)
Cigarettes a day in pregnancy	1.02 (2.05)	1.03 (2.18)	1 (1.73)	221.5 (*p* = .87)
Daily caffeine use (mg) in pregnancy	293.78 (223.01)	322.24 (247.53)	217.15 (111.63)	288.5 (*p* = .16)
Daily alcohol use (mg) in pregnancy	1.92 (2.94)	1.82 (2.88)	2.19 (3.19)	222 (*p* = .90)
Maternal BMI start pregnancy	21.24 (2.80)	21.22 (2.86)	21.19 (2.86)	0.036 (*p* = .97)
Maternal BMI end of pregnancy	26.15 (3.26)	25.99 (3.22)	26.45 (3.22)	-0.417 (*p* = .68)
	**N (%)**	**N (%)**	**N (%)**	Chi square test**
Highest level of education mother				*p* = .35
No High-School or Test Equivalent	6 (12.50)	3 (8.57)	3 (23.08)	
High School or Test Equivalent	11 (22.92)	7 (20.00)	4 (30.77)	
Undergraduate Level (Associate, Bachelor)	13 (27.08)	10 (28.57)	3 (23.08)	
Graduate Level (master, PhD.)	18 (37.50)	15 (42.86)	3 (23.08)	
Highest level education father				*p* = .76
No High-School or Test Equivalent	7 (14.58)	4 (11.43)	3 (23.08)	
High School or Test Equivalent	12 (25.00)	9 (25.71)	3 (23.08)	
Undergraduate Level (Associate, Bachelor)	6 (12.50)	5 (14.29)	1 (7.69)	
Graduate Level (Master, PhD.)	23 (47.92)	17 (48.57)	6 (46.15)	
Mothers employed or not				*p* = .66
Yes	41 (85.42)	29 (82.86)	12 (92.31)	
No	7 (14.58)	6 (17.14)	1 (7.69)	
Fathers employed or not				*p* = 1.00
Yes	46 (95.83)	33 (94.29)	13 (100.00)	
No	2 (4.17)	2 (5.71)	0 (0.00)	
Mother level of employment				*p* = .39
Unskilled or low skilled worker	9 (21.95)	5 (17.24)	4 (33.33)	
Skilled worker (e.g., technician, clerk)	4 (9.76)	2 (6.90)	2 (16.67)	
Highly skilled worker (e.g., civil servant, primary school teacher)	12 (29.27)	9 (31.03)	3 (25.00)	
Academic profession (e.g., higher civil servant, academic teacher)	16 (39.02)	13 (44.83)	3 (25.00)	
Father level of employment				*p* = .63
Unskilled or low skilled worker	15 (32.61)	9 (27.27)	6 (46.15)	
Skilled worker (e.g., technician, clerk)	3 (6.52)	3 (9.09)	0 ( 0.00)	
Highly skilled worker (e.g., civil servant, primary school teacher)	6 (13.04)	5 (15.15)	1 (7.69)	
Academic profession (e.g., higher civil servant, academic teacher)	22 (47.83)	16 (48.48)	6 (46.15)	
Married				*p* = .47
Yes	46 (95.83)	34 (97.14)	12 (92.31)	
No	2 (4.17)	1 (2.86)	1 (7.69)	
**28 years old offspring**	**Follow-up sample (n = 49) ** **Mean (SD)**	**Low-Medium anxiety (LMA) group (n = 36)** **Mean (SD)**	**High-anxiety (HA) group (n = 13)** **Mean (SD)**	Independent sample t-test*
Birth weight	3214.69 (559.03)	3290.28 (490.6)	3005.38 (695.31)	-1.60 (*p* = .12)
Gestational age at birth	272.29 (12.8)	273.61 (11.76)	268.62 (15.24)	266 (*p* = .48)
Birth weight adapted for gestational age	0.01 (1.02)	0.08 (0.87)	− 0.19 (1.4)	0.82 (*p* = .42)
Age 28 follow-up (age when tested)	27.84 (0.43)	27.75 (0.39)	28.08 (0.45)	**136.5 (*p*** **= .03)**
	**N (%)**	**N (%)**	**N (%)**	Chi square test**
Highest level of education offspring				*p* = .62
No High-School or Test Equivalent	0 (0)	0 (0)	0 (0)	
High School or Test Equivalent	2 (4.25)	1 (2.86)	1 (8.33)	
Undergraduate Level (Associate, Bachelor)	18 (38.30)	14 (40.00)	4 (33.33)	
Graduate Level (Master, PhD.)	27 (57.45)	20 (57.14)	7 (58.33)	
Offspring level of employment				*p* = .72
Unskilled or low skilled worker	0 (0)	0 (0)	0 (0)	
Skilled worker (e.g., technician, clerk)	2(4.54)	1 (3.13)	1 (7.69)	
Highly skilled worker (e.g., civil servant, primary school teacher)	17(38.64)	13 (40.62)	4 (30.77)	
Academic profession (e.g., higher civil servant, academic teacher)	25 (56.82)	18 (56.25)	7 (53.84)	
Offspring employed or not				*p* = .56
Yes	44(93.62)	32 (91.43)	12 (100.00)	
No	3(6.38)	3 (8.57)	0 (0.00)	
Psychiatric diagnoses, lifetime				
Major Depressive Disorder	1 (2.00)	1 (2.90)	0 (0.00)	*p* = .54
Anxiety Disorder (Panic, GAD, Social)	5 (10.2)	3 (9.70)	2 (15.40)	*p* = .59

Note: *Non-parametric analysis by Wilcox Rank Sum test was performed if assumptions for t-test were not met

** Non-parametric analysis by Fisher’s exact test was performed if assumptions for Pearson’s Chi-squared test were not met

Notes: it is possible that some cases are missing data across the variables; summary statistics calculated on available data only. One twin was included in our sample, therefore sample size of LMA parents is 35 instead of 36. GAD = Generalized Anxiety Disorder

### Materials

#### Maternal anxiety during pregnancy

To investigate the anxiety level of the mothers during pregnancy, the Dutch version of the State Trait Anxiety Inventory (STAI) (Van der Ploeg et al., 1980) was used. Two offspring anxiety subgroups were defined, “High anxiety” (HA; n = 13) group versus “low-to-medium anxiety” (LMA; n = 36) group, based on the mother’s STAI state anxiety subscale total score during week 12–22 week of pregnancy. The threshold of the dichotomous split between the group is 43 (i.e., percentile 75 in a reference population (Van der Ploeg et al., 1980), which has been used in different cohorts (Koelewijn et al., 2017; Mennes et al., 2006; van den Heuvel et al., 2018b). As expected, mean maternal anxiety was higher in HA group (M_HA_= 50.29, *SD* = 6.44) compared to LMA group (M_LMA_ = 34.59, *SD* = 4.73; *t* = -9.24, *p* < .001) and are situated at respectively decile 9 versus decile 5 of a Dutch non-clinical community sample described in the STAI Manual (Van der Ploeg et al., 1980).

#### Covariates

Postnatally, mothers completed the STAI when the child was 1, 10, and 28 weeks old (postnatal part of first wave), at 8/9 years (second wave), at 14/15 years (third wave), at 17 years (fourth wave) and at 20 years (fifth wave). In a principal component analysis conducted on the postnatal trait anxiety measures obtained in wave one to four, the first component explained 66.2% of the variance. This standardized component score was created for each mother, and labeled “postnatal anxiety”. Given that postnatal experience and other potential confounds, i.e., alcohol and caffeine use, smoking, gestational age at birth and birth weight, could affect neurobehavioral outcomes of the child, all of these were recorded and examined. These demographics and potential confounds (alcohol and caffeine use, smoking, and offspring gestational age at birth and birth weight corrected for gestational age, maternal/paternal age, postnatal anxiety) were not significantly different between LMA and HA group after correction for multiple testing with Bonferoni correction (Table 1).

#### Offspring MRI and fMRI Data Acquisition and Processing

##### Data acquisition

MR-scans were acquired using a Philips Achieva 3T scanner (Philips, Best, The Netherlands) with 32 channel head coil and in-coil AC/DC conversion (dStream). Functional images for rs-fcMRI were obtained with a T2*-weighted echo-planar imaging (EPI) sequence (4 × 4 × 4 mm3, TE/TR = 33 ms/1700 ms; FOV = 230 × 120 × 230 mm; 4 mm slice thickness, 30 slices, 7 min acquisition time; 250 volumes). For the acquisition of this scan, participants were requested to relax, but not to fall asleep. For anatomical mapping and optimal registration to standard space, a T1-weighted image was acquired (MPRAGE, resolution 1 × 1 × 1 mm3, TE/TR = 4.6ms/9.6 ms, FOV = 192 × 250 × 250, 1.2 mm slice thickness, 160 slices, 6 min acquisition time).

#### MRI processing

Preprocessing followed established procedures for functional imaging using FSL software (Smith et al., 2004), see **Supplemental materials**. After preprocessing, functional connectivity was analyzed based on a 32 by 32 matrix of partial correlations between regional BOLD signals. The nodes of interest in this study were all 32 cortical and cerebellar regions (or ROIs) from the network atlas as provided by the Conn toolbox (atlas is based on 497 subjects from the Human Connectome Project (http://www.humanconnectome.org), see **Table S2** in supplement for a description of the regions). To estimate the functional connectivity between 32 nodes, partial correlations, i.e., corrected for the remaining connections to avoid spurious effects in network modeling, were calculated using the Conn toolbox (Whitfield-Gabrieli & Nieto-Castanon, 2012) implemented in MATLAB (http://www.nitrc.org/projects/conn; version 17.f).

### Statistical analyses

First, rsFC partial correlations between the 32 nodes of interest (as defined by the atlas in the Conn toolbox) were compared between the HA and LMA groups using ANCOVAs, i.e., adjusted for sex, birth weight (corrected for gestational age), and postnatal anxiety, since these variables may influence rsFC. These comparisons were False Discovery Rate (FDR-) corrected for multiple comparisons at *p* < .05 (FDR correction for 32*32 comparisons).

Second, Network Based Statistics (NBS; Zalesky et al., 2010) as part of the Conn toolbox was implemented to compute functional network differences between the HA and LMA groups, using a different (more liberal) approach to correct for multiple comparisons. The first step in the NBS required a threshold based on a significant test statistic for each connection based on the HA and LMA group difference (i.e., the significant test statistic here was the uncorrected *p* < .05 of the ANCOVA as computed for each ROI-to-ROI connection). The second step included cluster-based permutation testing (randomizing group assignment) to determine whether the subnetwork is significantly larger than chance (NBS seed-based threshold, *p* < .05, FDR-corrected). Due to the small sample of our cohort, the outcome of the NBS will be interpreted with care and will mainly serve as a sensitivity analysis of the ANCOVA.

Third, graph theoretical network metrics were computed for each individual participant for two different atlases. For the first parcellation, brain regions were selected using the 32 cortical and cerebellar regions (or ROIs) from the network atlas as provided by the Conn toolbox. For the second parcelation, we used 68 cortical regions of the FreeSurfer’s Desikan Killiany atlas. Individual weighted graphs were thresholded (threshold = 0.3, only positive connections > 0.30 ) for both atlases. Individual whole-brain graph metrics included density, connectivity strength, global clustering, normalized global clustering, global efficiency, normalized global efficiency and normalized small worldness and were computed using the Brain Connecitvity Toolbox implemented in MATLAB (BCT; Rubinov and Sporns, 2010). Normalized graph metrics were obtained by taking the ratio of the actual graph metrics and graph metrics observed in the 1000 random networks. LMA and HA group differences of graph metrics were determined using ANCOVAs, i.e., adjusted for sex, birth weight, and postnatal anxiety.

## Results

### Group based functional connectomes

**Fig. 1:**
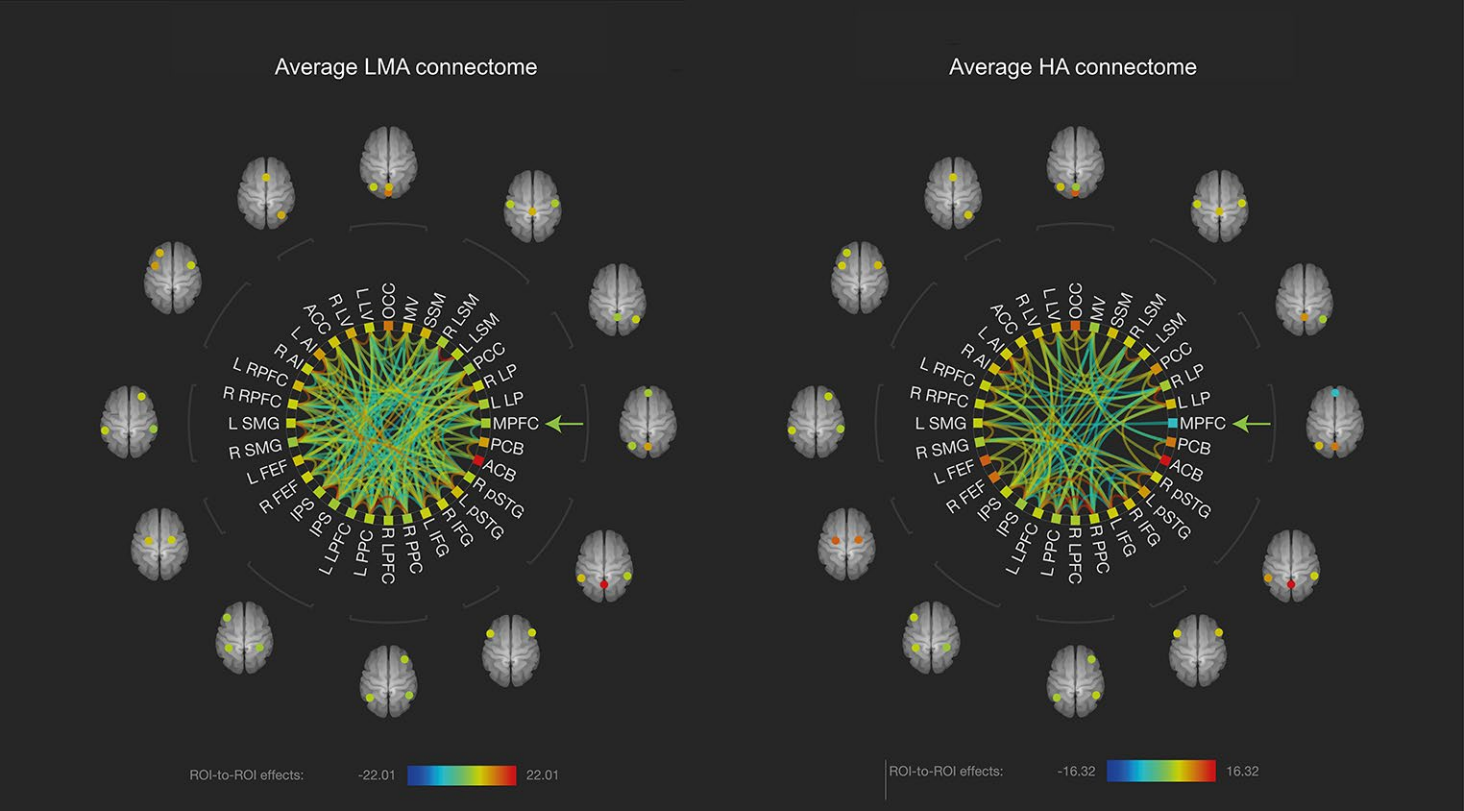
**Group-based functional connectomes.** Figure displays the functional group-based connectome rings of offspring of low-medium (left) and high (right) prenatal anxiety. Significant ROI-to-ROI connections (p < .05, FDR-corrected) of both groups are displayed in a color ranging from blue (negative) to red (positive) and represent the T-statistics (see color-bar below the connectome graphs for specific values). The arrow points to the MPFC ROI to indicate that this region (visually) shows most difference between groups, with less significant connections to other areas in the HA group as compared to the LMA group. ROI labels and descriptions for the abbreviations can be found in the supplemental materials, **Table S2**

We examined the functional connectome of the two groups (LMA versus HA) by plotting significant ROI-to-ROI connections (*p*_*FDR*_ < 0.05) per group (see Fig. 1). Based on this Figure, the HA group shows weaker overall connectivity compared to the LMA group, indicated by the lower number of significant connections in the HA group. More specifically, the medial prefrontal cortex (MPFC) seems at the core of the weaker connectivity in the HA group, showing less significant connections in the HA group as compared to the LMA group.

### Association between maternal anxiety in pregnancy and offspring rsFC

**Fig. 2:**
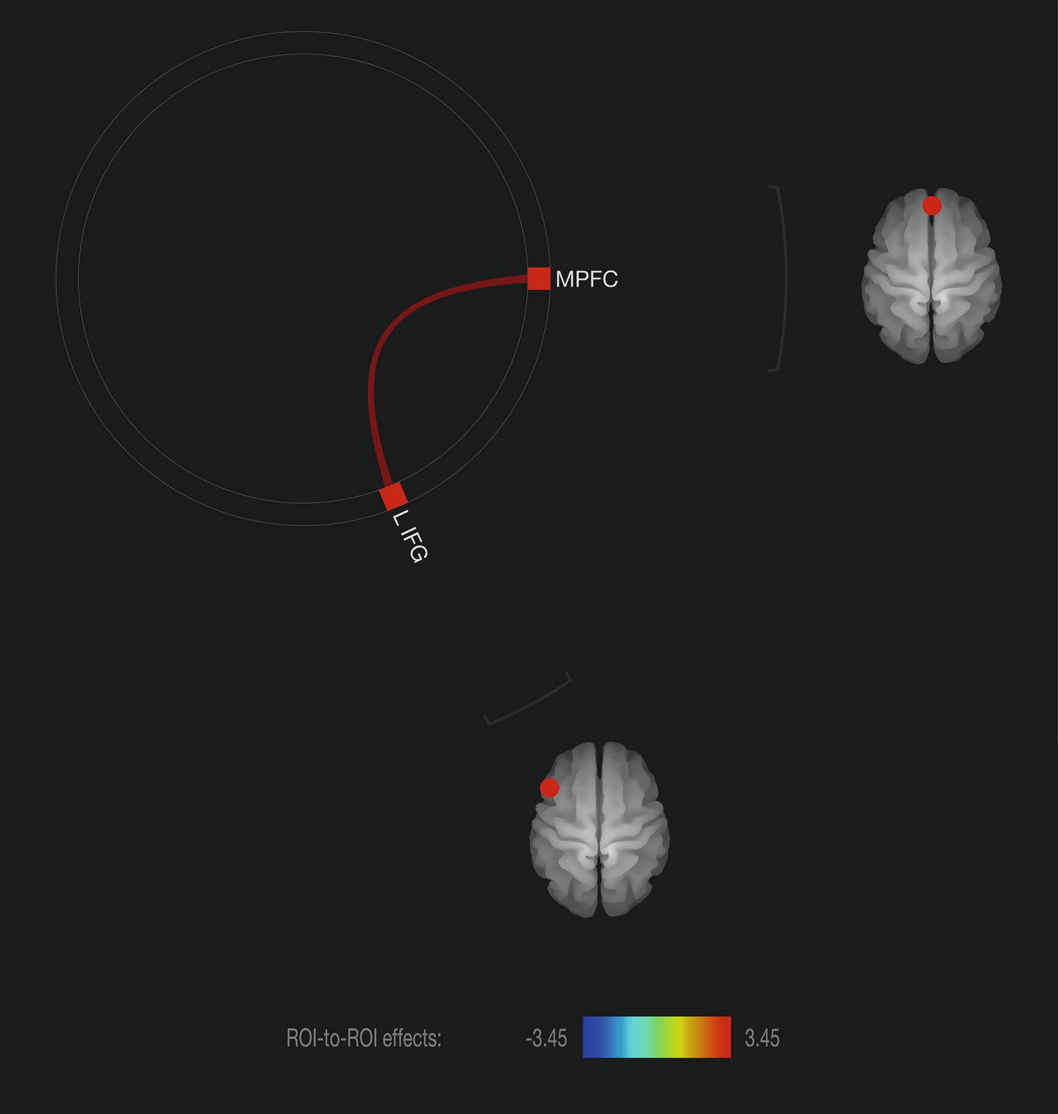
**Functional connectivity group-comparison.** Significant group-differences of adult offspring exposed to low-medium maternal anxiety (LMA) and high maternal anxiety (HA) in functional ROI-to-ROI connectivity (*p* < .05, FDR-corrected). Connections and nodes are displayed in red (positive), and represent the T-statistics (see color-bar for specific values). Left cortical regions are displayed on the left of the brain

Results of the ANCOVA group comparison, corrected for sex, birth weight (adapted to gestational age), and maternal postnatal anxiety, yielded a significant difference in connectivity between MPFC and left inferior frontal gyrus (IFG) (*t =* 3.45, *p*_*uncorr*_ = 0.0012, *p*_*FDR*_ = 0.0383). More specifically, this positive correlation was stronger in the LMA group (for visualization see Fig. 2). This finding is in line with our observations based on Fig. 1. The effect size of prenatal anxiety on the connectivity strength between MPFC and left IGF was *R*^*2*^ = 0.18, indicating that 18% of the variance in the offspring’s connectivity was explained by maternal prenatal anxiety. Moreover, the effect size of the covariates alone on the connectivity strength between MPFC and left IGF was *R*^*2*^ = 0.08. The effect size became significantly larger when prenatal anxiety was added to the model with covariates, *R*^*2*^ = 0.28 (Δ*R*^*2*^ = 0.20). A boxplot of the two connectivity distributions can be found in **Supplemental Figure**S1. Additional to the group comparison, we examined the linear association between connectivity strength (between MPFC and left IFG) and prenatal anxiety using the continuous STAI-scores. The analysis is in line with the original ANCOVA and revealed a negative association between prenatal anxiety and MPFC and left IFG connectivity strength (bivariate Pearson’s *r* = -.30, *p* = .035).

Additional analyses were performed to examine the possible influence of maternal postnatal anxiety as driving factor. The Variance inflation Factor (VIF) for prenatal anxiety and each predictor variable (sex, postnatal anxiety and birth weight adapted to gestational age) was low (*VIF* < 1.31) and the correlation between pre- and postnatal anxiety was only low to moderate (*R* = .467, *p* < .001). Therefore, we assumed that multicollinearity was not an issue. We conducted an additional ANCOVA to make sure that the relationship between prenatal anxiety and offspring FC outcome was not conditioned on postnatal anxiety. ANCOVA results (corrected for sex and adapted birth weight only) remained significant without controlling for maternal postnatal anxiety (MPFC and left IFG, *t* = 3.54, *p*_*uncorr*_ = 0.0009, *p*_*FDR*_ = 0.0288).

### Network-based statistics

**Fig. 3:**
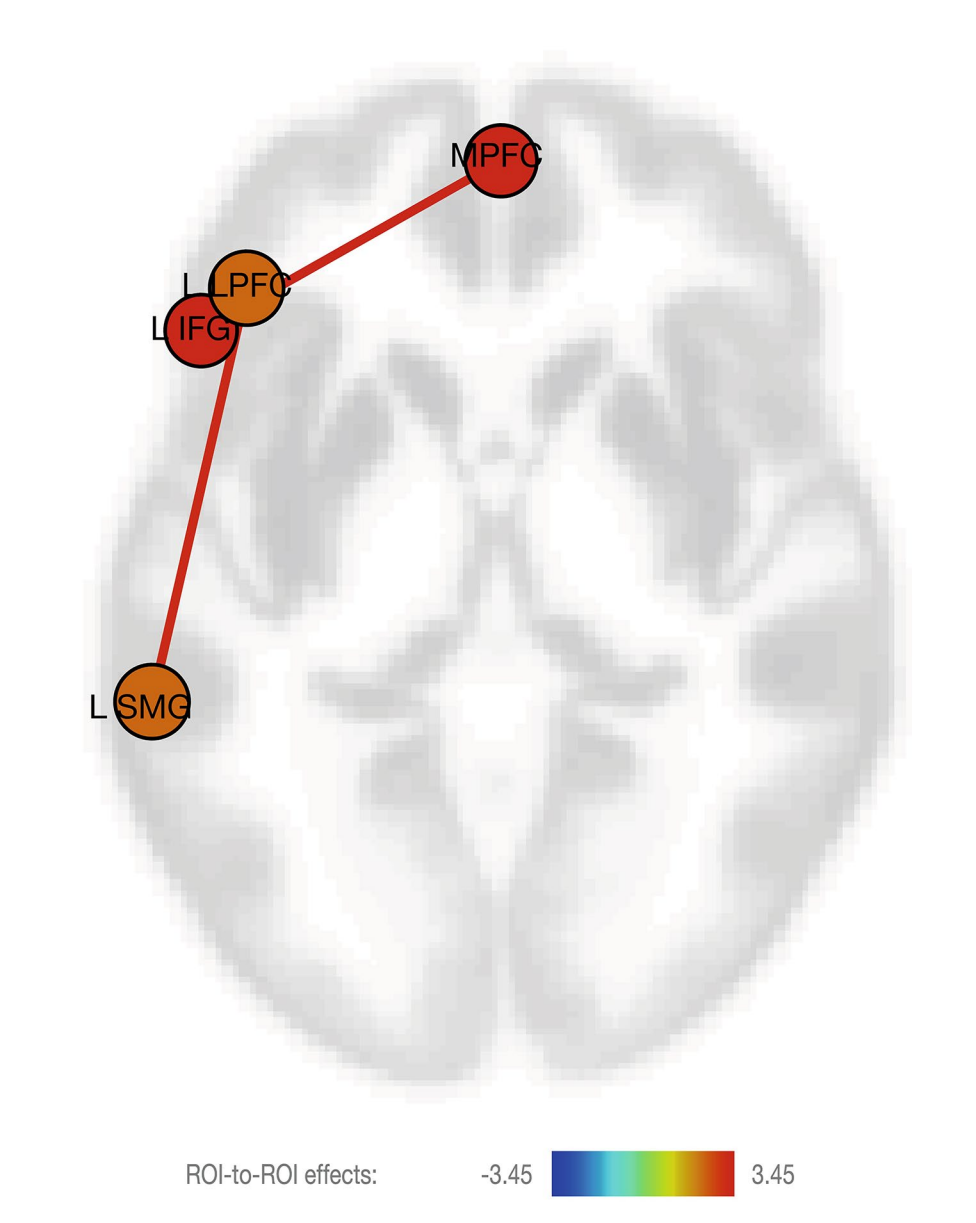
**Functional connectivity group-comparison using network-based statistics.** Significant functional ROI-to-ROI connectivity (p_uncorr_ <0.01, and NBS, seed-based threshold of p < .05_FDR_) group-differences of adult offspring that exposed to low-medium maternal anxiety (LMA) and high maternal anxiety (HA). Connections are displayed in red (positive) and color of the nodes represent the T-statistics (see color-bar below the plot for specific values). Connections are presented on an axial view of an average T1 brain. Significant clusters can be found between medial prefrontal cortex (MPFC) and left inferior frontal gyrus (L IFG) and between left prefrontal cortex (L PFC) and left supramarginal gyrus (L SMG). The figure represents an axial MRI slide of the brain, left cortical regions are displayed on the left of the cortex

The ANCOVA group comparison, including a network-based statistic threshold, yielded similar findings with a significant group difference in network-connectivity between medial prefrontal cortex (MPFC) and left inferior frontal gyrus (IFG) (*p*_*uncorr*_ = 0.0012, *p*_FDR_ <0.05). We observed additional effects; i.e., we also observed a significant group difference in network-connectivity between left lateral prefrontal cortex (LPFC) and left somatosensory motor gyrus (SMG) (*p*_*uncorr*_ = 0.0015, *p*_FDR_ <0.05). These positive correlations both showed to be stronger in the LMA group, for visualization see Fig. 3.

### Group differences in graph metrics

In contrast to the ROI-to-ROI analyses, no group (HA versus LMA) differences were found for global network-based density, connectivity strength, global clustering, normalized global clustering, global efficiency, normalized global efficiency and normalized small worldness (all *p’s* > 0.05, see **Table S3** for more details). Results remained non-significant when controlled for covariates sex, birth weight (adapted to gestational age), and maternal postnatal anxiety (all *p’s* > 0.05, see **Table S3** for more details).

## Discussion

This study demostrated that, in specific networks, adult functional brain connectivity is weaker in adults exposed to higher maternal anxiety at 12–22 weeks of gestation, compared to adults exposed to low to medium maternal anxiety in that period. This association was shown in a prospective prenatal cohort with a postnatal follow-up of 28 years, indicating a long-term effect of prenatal stress exposure on functional network connectivity. In the analyses performed, differences were most pronounced for the medial prefrontal cortex (MPFC), showing weaker functional connectivity in prenatally exposed adult offspring. Specifically, we found weaker functional connectivity between MPFC and the left inferior frontal gyrus (IFG) and between the left lateral prefontal cortex (LPFC) and the left somatosensory motor gyrus (SMG). By contrast, global brain alterations, as measured with graph metrics, did not emerge from our data. This may indicate specific alterations of weaker frontal brain connectivity, instead of a weaker connectivity throughout the brain in adult offspring of mothers with high anxiety in pregnancy. Also of interest is the observed laterality in effect, with all findings presenting in the left hemisphere.

Our finding of lower functional connectivity of the MPFC and left PFC with other brain regions is in line with earlier findings of prenatal distress follow-up studies using brain imaging techniques. Firstly, our results are in line with a very recent study from the FinnBrain Birth Cohort study: Rajasilta et al. (2023) also reported altered MPFC functional connectivity in neonates prenatally exposed to maternal stress. Secondly, our results are in line with the finding that offspring of women (highly) psychologically distressed during pregnancy, show altered structural or functional connectivity of the prefrontal cortex with other brain areas (Hay et al., 2020; Humphreys et al., 2020; Qiu et al., 2015; Soe et al., 2018). Most of these studies focus their examination on rsFC of the (pre)frontal region with limbic structures and were conducted in neonates, infants and children only. Moreover, multiple studies linked prenatal maternal distress to behavioral dysregulation (DiPietro et al., 2002), enhanced vigilance (van den Heuvel et al., 2015; van den Heuvel, Henrichs, Heuvel et al., 2018a, b), and executive dysfunction (Buss et al., 2011; Pearson et al., 2016), which indirectly suggests frontal neural changes (McKlveen et al., 2015). Lastly, autonomic, motor, emotional and neurocognitive problems found in previous waves of our offspring cohort, could indirectly be linked to altered (pre)frontal functional connectivity. In the cohort being examined in the current study, exposure to high maternal anxiety in pregnancy was associated with the following observations that indirectly reflect alterations in early neurodevelopment: altered fetal and neonatal sleep-wake cycles (Van den Bergh, 1990), lower scores on subscales of the Wechsler Intelligence Scale for children (WISC)-III at ages 14–15 years, deficits in endogenous cognitive control/sustained attention measured with specific cognitive tasks at age 14–15 (Van den Bergh et al., 2005, 2006) and 17 years (Mennes et al, 2006), which were corroborated by results of functional brain imaging measures, i.e., task-related EEG at age 17 years (Mennes et al., 2009) and functional MRI measures at age 20 years (Mennes et al., 2020). Behavioral (regulation) problems found in previous waves of our offspring cohort included crying, eating and sleep problems, and difficult temperament in infancy (Van den Bergh, 1990), ADHD, impulsivity, and externalizing problems in childhood and early adolescence (Van den Bergh & Marcoen, 2004; Van den Bergh et al., 2005). Still, future longitudinal investigations in a different (larger) cohort, preferably with repeated testing with neuroimaging methods at consecutive ages, should replicate our findings.

It is challenging to interpret the strength of our observed effects (18% explained variance) in the context of previous findings in the literature, given the small number of studies with similar research questions and sample. Additionally, we noticed that many articles do not report effect sizes. A few related papers did report effect sizes: between 6 and 13% explained variance in infant ERP responses by maternal anxiety and mindfulness during pregnancy (van den Heuvel et al., 2015), 6% explained variance in newborn hippocampal volume by maternal psychological stress during pregnancy (Moog et al., 2021), and around 2% explained variance in fetal connectome by maternal anxiety during pregnancy (De Asis-Cruz et al., 2020). A better comparison may be the study by Mareckova et al. (2020), who examined the effect of prenatal maternal depression on the structural brain age of young adult offspring. They found an effect size of 6% explained variance. Since we found an effect size of 18% explained variance, it seems that our observed effect size is relatively large, in comparison with similar studies.

The finding of exclusive effects for the left hemisphere could point to lateralization of effect. Some other studies have also reported results in the left hemisphere only. For instance, in a recent study, Moog et al. (2021) reported smaller volumes of the left hippocampus, but not the right, in infants prenatally exposed to higher levels of maternal perceived distress during pregnancy. Additionally, recent work with fetal imaging showed decreased cerebellar-insular functional connectivity in fetuses of distressed mothers, for the left insula only (van den Heuvel et al., 2021). Given that the left hemisphere may develop relatively faster than the right hemisphere during the prenatal period (Andescavage et al., 2017), it could be more sensitive to prenatal environmental insults such as maternal distress. Interestingly, Vasung et al. (2020) specifically reported that the left IFG – a key region that came up in our results – has a faster volume growth than the right IFG, potentially making it more vulnerable. Yet, most human studies do not discuss laterality effects and no human study to date has specifically focused on laterality effects of prenatal stress exposure, nor its potential mechansims.

The current study has several strengths and limitations. A first evident strength is the prospective design and the follow-up study spanning almost 29 years with an offspring response rate as high as 60% at ag 28 years. Second, the proportion of pregnant women experiencing high levels of state anxiety was relatively high; 34% had a score of > 43 at 12–22 weeks of pregnancy, which is a prerequisite for revealing, if any, effects of high anxiety. Nevertheless, our study also has several limitation that should be noted. A first limitation of our study is the relatively small size of the sample (N = 49). This did not allow us to conduct further analyses on specific sex-interactions, which could have been interesting. Second, the acquisition time of 7 min for this study was relatively short. Research has been shown that the reliability and similarity can be greatly improved by increasing the scan lengths up to 13 mintes (Birn et al., 2013). Third, this study did not include any physiological markers of distress/anxiety of the mother. We focused on the subjective, self-reported experience of the mother, rather than biological markers. However, since offspring outcome measures were biological markers (i.e. brain imaging) and not maternal reported measures, shared method variance inflating the associations is not at stake. When using subjective, mother-reported outcome measures, mothers who were anxious during pregnancy may have a biased perception of the behavior of their child (usually more negative, see Pesonen et al., 2005). Fourth, no genetic sensitive design was used and, therefore, we cannot rule out genetic mechanisms at play. However, previous research examining the effect of prenatal exposure to objective, random stressors, such as natural disasters, have shown that genetic mechanisms cannot (only) explain the observed effects of stress exposure on the offspring’s brain (Jones et al., 2019).

## Conclusions

Our findings suggest that the adult connectome may be influenced by the prenatal environment. However, replication in larger samples is necessary to confirm this tentative conclusion. Although the brain architecture continues to show plasticity throughout adult life, some biological changes that compromise flexible adaptation and resilience might already be laid down early in life (McEwen et al., 2015). Our current rsFC results indicate that individuals exposed to varying levels of maternal anxiety at 12 to 22 weeks of pregnancy show weaker functional brain connectivity of the medial prefrontal cortex (MPFC) and left prefontal cortex (LPFC) with some other brain regions in the left hemisphere, emphasizing an altered frontal neural network in the adults who were prenatally exposed to high maternal anxiety. Such alterations in frontal connectivity may put these adults at higher risk for specific cognitive deficits and executive dysfunctions, mental health issues, and potentially even neurodegenerative disorders in later life (Faa et al., 2014). Future work may seek to replicate our rsFC finding and try to characterize the potential neural vulnerability of prenatally exposed individuals better, e.g., based on properties of dynamic fluctuations in whole-brain rsFC analyses and/or task-related fMRI analyses.

### Electronic supplementary material

Below is the link to the electronic supplementary material.

Supplementary Material 1

## Data Availability

The data that support the findings of this study are available from the corresponding author on reasonable request.
