# Supplementary material for: Maternal anxiety during pregnancy is associated with weaker prefrontal functional connectivity in adult offspring
Source: Brain Imaging Behav. 2023 Jun 29;17(6):595–607. doi: 10.1007/s11682-023-00787-1 (PMC10733226; doi:10.1007/s11682-023-00787-1)
Supplement: Supplementary file 1 — Supplementary Material 1 [file 11682_2023_787_MOESM1_ESM.docx]

**Maternal anxiety during pregnancy is associated with weaker prefrontal functional connectivity in adult offspring**

*Elise Turk^1a^, Marion I. van den Heuvel^1a*^, Charlotte Sleurs^1,2^, Thibo Billiet^3^, Anne Uyttebroeck^2^, Stefan Sunaert^4^, Maarten Mennes^5^, Bea Van den Bergh^6,7^*

^a^Authors share first authorship

*^1^Department of Cognitive Neuropsychology, Tilburg University. The Netherland; email:* [*m.i.vdnheuvel@tilburguniversity.edu*](mailto:m.i.vdnheuvel@tilburguniversity.edu)*;* [*e.turk@tilburguniversity.edu*](mailto:e.turk@tilburguniversity.edu)

*^2^ Department of Oncology, Catholic University of Leuven, KU Leuven, Leuven, Belgium*

*e-mail:* [*charlotte.sleurs@kuleuven.be*](mailto:charlotte.sleurs@kuleuven.be)*;* [*anne.uyttebroeck@uzleuven.be*](mailto:anne.uyttebroeck@uzleuven.be)

*^3^ Icometrix, Leuven, Belgium; e-mail:* [*thibo.billiet@icometrix.com*](mailto:thibo.billiet@icometrix.com)

^4^ *Radiology, KU Leuven, Belgium; e-mail* [*stefan.sunaert@uzleuven.be*](mailto:stefan.sunaert@uzleuven.be)

*^5^ Donders Institute for Brain, Cognition and Behaviour, Radboud University Nijmegen, The Netherlands; e-mail: m.mennes@donders.ru.nl*

*^6^ Health Psychology Research Group, Catholic University of Leuven, KU Leuven, Leuven, Belgium; e-mail: bea.vandenbergh@kuleuven.be*

*^7^ Department of Welfare, Public Health and Family, Flemish Government, Brussels, Belgium*, e-mail: [*bea.vandenbergh@vlaanderen.be*](mailto:bea.vandenbergh@vlaanderen.be)

**Supplemental methods**

**Supplemental demographics**

At 12-22 weeks of pregnancy, mothers of the follow-up sample had a mean age of 26.19 years (*SD*=2.55) and fathers of 28.32 years (*SD*=2.55). Almost all mothers were living together, were married (95.83%) and had a stable relationship (*Mean* =36.50 months; *SD*=25.67). The percentage of parents and offspring being gainfully employed was very high (mothers: 85.42%; fathers: 95.83; offspring: 88.89%) and they were highly educated as between 60% and 96% (mothers: 64.58%; fathers: 60.42%; offspring: 95.75%) obtained an undergraduate (associate or bachelor) or graduate (master or Ph.D.) level degree, respectively (see **Table 1)**.

Additionally, we examined whether there were any significant differences between the follow-up study (n = 48 mothers/49 offspring, one twin) and the dropout sample (n = 38 mothers/39 offspring, one twin), see **Table S1**. After Bonferonni correction, no differences between the follow-up and dropout sample emerged (see **Table S1** for details). We examined whether there were any differences in parental demographics between the participants versus the drop-out group using Pearson’s chi-square tests or Fisher’s exact tests. There were no important differences, be it that maternal age and social class were slightly higher (as more fathers were employed at the highest level) in the current sample (see supplemental **Table S1**). **Table S1** shows supplemental demographics and descriptive statistics between resting state connectivity follow-up study (n = 48 mothers/49 offspring), the dropouts (n = 38 mothers/39 offspring), and the full sample (n = 86 mothers/88 offspring). Importantly, maternal anxiety levels during pregnancy were not different between the current sample and those lost to follow-up. We then explored whether there were any important sex differences in maternal state anxiety, birth weight, and postnatal anxiety, in our sample using Independent Samples t-tests. No sex differences emerged on any predictor or covariate.

**Table S1.** Demographics and descriptive statistics between resting state connectivity follow-up study, the dropouts, and the full sample

| **Variables** | **Full sample (n=86)** | **Follow-up sample (n=48)** | **Dropout sample (n=38)** | **Follow-up vs dropout t-test**** |
| --- | --- | --- | --- | --- |
| **Parents** | **Mean (SD)** | **Mean (SD)** | **Mean (SD)** |  |
| Maternal state anxiety 12-22 weeks of pregnancy | 39.29 (8.54) | 38.84 (8.74) | 39.86 (8.37) | *979 (p=.56)* |
| Postnatal maternal trait anxiety | -.01 (1.00) | .12 (.99) | -.24 (.98) | *-1.53 (p=.13)* |
| Maternal age at 12-22 weeks, years | 25.64 (2.79) | 26.19 (2.55) | 24.95 (2.96) | *-2.09 (p=.04)* |
| Paternal age at 12-22 weeks, years | 27.86 (3.90) | 28.32 (4.16) | 27.2 (3.44) | *551.5 (p=.23)* |
| Social class (based on education both parents) | -.02 (1.01) | .20 (.99) | -.30 (.99) | *641 (p=.02)* |
| Months married | 36.77 (25.88) | 36.5 (25.67) | 37.16 (26.59) | *752.5 (p=.87)* |
| Cigarettes a day in pregnancy | 1.50 (3.70) | 1.02 (2.05) | 2.11 (5.04) | *948.5 (p=.70)* |
| Daily caffeine use (mg) in pregnancy | 304.83 (244.04) | 293.78 (223.01) | 318.79 (270.72) | 906 *(p=.96)* |
| Daily alcohol use (mg) in pregnancy | 2.34 (5.04) | 1.92 (2.94) | 2.87 (6.85) | 992 (*p=.47*) |
|  | **N (%)** | **N (%)** | **N (%)** | **Chi square test***** |
| Highest level of education mother |  |  |  | *p=.06* |
| No High-School or Test Equivalent | 16 (18.60) | 6 (12.50) | 10 (26.32) |  |
| High School or Test Equivalent | 22 (25.58) | 11 (22.92) | 11 (28.95) |  |
| Undergraduate level (associate, bachelor) | 25 (29.07) | 13 (27.08) | 12 (31.58) |  |
| Graduate level (master, PhD.) | 23 (26.74) | 18 (37.50) | 5 (13.16) |  |
| Highest level education father |  |  |  | *p=.15* |
| No High-School or Test Equivalent | 13 (15.29) | 7 (14.58) | 6 (16.22) |  |
| High School or Test Equivalent | 26 (30.59) | 12 (25.00) | 14 (37.84) |  |
| Undergraduate level (associate, bachelor) | 14 (16.47) | 6 (12.50) | 8 (21.62) |  |
| Graduate level (master, PhD.) | 32 (37.65) | 23 (47.92) | 9 (24.32) |  |
| Mothers employed or not |  |  |  | *p=.06* |
| Yes | 67 (77.91) | 41 (85.42) | 26 (68.42) |  |
| No | 19 (22.09) | 7 (14.58) | 12 (31.58) |  |
| Fathers employed or not |  |  |  | *p=1.00* |
| Yes | 82 (96.47) | 46 (95.83) | 36 (97.30) |  |
| No | 3 (3.53) | 2 (4.17) | 1 (2.70) |  |
| Mother level of employment |  |  |  | *p=0.10* |
| Unskilled or low skilled worker | 14 (20.90) | 9 (21.95) | 5 (19.23) |  |
| Skilled worker (e.g., technician, clerk) | 11 (16.42) | 4 (9.76) | 7 (26.92) |  |
| Highly skilled worker (e.g., civil servant, primary school teacher) | 22 (32.84) | 12 (29.27) | 10 (38.46) |  |
| Academic profession (e.g., higher civil servant, academic teacher) | 20 (29.85) | 16 (39.02) | 4 (15.38) |  |
| Father level of employment |  |  |  | *p=.01* |
| Unskilled or low skilled worker | 25 (30.49) | 15 (32.61) | 10 (27.78) |  |
| Skilled worker (e.g., technician, clerk) | 14 (17.07) | 3 (6.52) | 11 (30.56) |  |
| Highly skilled worker (e.g., civil servant, primary school teacher) | 13 (15.85) | 6 (13.04) | 7 (19.44) |  |
| Academic profession (e.g., higher civil servant, academic teacher) | 30 (36.59) | 22 (47.83) | 8 (22.22) |  |
| Married |  |  |  | *p=1.00* |
| Yes | 81 (95.29) | 46 (95.83) | 35 (94.59) |  |
| No | 4 (4.71) | 2 (4.17) | 2 (5.41) |  |
| **Offspring** | **Full sample (n=88)**  **Mean (SD)** | **Follow-up sample (n=49)**  **Mean (SD)** | **Dropout sample (n=39)**  **Mean (SD)** | **Follow-up vs dropout t-test**** |
| Birth weight | 3223.59 (628.47) | 3214.69 (559.03) | 3235.69 (720.48) | 989.5 (*p = .34*) |
| Gestational age at birth | 272.67 (13.64) | 272.29 (12.8) | 273.19 (14.85) | 1017 (*p=.*34) |
| Birth weight adapted for gestational age | 0 (1) | .01 (1.02) | -.02 (0.98) | -.155 (*p=.88*) |
|  | **N (%)** | **N (%)** | **N (%)** | **Chi square test***** |
| Highest level of education offspring |  |  |  | *p=.62* |
| No High-School or Test Equivalent | 0 (0) | 0 (0) | 0 (0) |  |
| High School or Test Equivalent | 2 (4.25) | 1 (2.86) | 1 (8.33) |  |
| Undergraduate Level (Associate, Bachelor) | 18 (38.30) | 14 (40.00) | 4 (33.33) |  |
| Graduate Level (Master, PhD) | 27 (57.45) | 20 (57.14) | 7 (58.33) |  |
| Offspring level of employment |  |  |  | *p=.72* |
| Unskilled or low skilled worker | 0 (0) | 0 (0) | 0 (0) |  |
| Skilled worker (e.g., technician, clerk) | 2(4.54) | 1 (3.13) | 1 (7.69) |  |
| Highly skilled worker (e.g., civil servant, primary school teacher) | 17(38.64) | 13 (40.62) | 4 (30.77) |  |
| Academic profession (e.g., higher civil servant, academic teacher) | 25 (56.82) | 18 (56.25) | 7 (53.84) |  |
| Offspring employed or not |  |  |  | *p=.56* |
| Yes | 44(93.62) | 32 (91.43) | 12 (100.00) |  |
| No | 3(6.38) | 3 (8.57) | 0 (0.00) |  |

*Notes*. Uncorrected p-values were presented;

*Two mothers had twins; in the follow-up study only one twin participated

**Non-parametric analysis by Wilcox Rank Sum test was performed if assumptions for t-test were not met; Bonferoni corrected p-values were used (*p*=0.05/12=0.004).

*** Non-parametric analysis by Fisher's exact test was performed if assumptions for Pearson's Chi-squared test were not met; Bonferoni corrected p-values were used (*p*=0.05/11=0.005).

Note: it is possible that some cases were missing data across the variables; summary statistics were calculated on available data only. Missing values of alcohol, caffeine use and smoking were imputed using weighted predictive mean matching using the MICE (version 2.9.0) algorithm in R for a more realistic imputation due to the skewed distribution of these variables (van Buuren and Groothuis-Oudshoorn, 2011).

***MRI preprocessing***

Preprocessing followed established procedures for functional imaging including realignment for head motion, grand mean scaling and spatial smoothing with gaussian kernel of 5 mm FWHM using FSL software (Smith et al., 2004). Root Mean Square (RMS) motion parameters were calculated for all subjects, in order to detect extreme movements. The distribution of RMS showed two outliers with RMS values > 1. These subjects were removed from the dataset for resting state analyses (n=2). Next, FSL’s melodic was applied to extract independent data components and ICA-AROMA (Pruim et al., 2015) was applied to identify and remove secondary effects of head motion. Finally, a temporal high-pass filter with 0.01 Hz cut-off was applied to remove scanner drifts. Signals extracted from a white matter mask and a CSF mask were regressed out using individual level GLMs. The residuals of this analysis were used for further connectivity analyses.

We obtained the transformation of the fMRI data to the participant’s high-resolution T1 anatomical space using FSL’s Boundary-Based Registration tool. A transformation from the participant’s T1 space to MNI152 standard space was obtained using linear alignment via FSL FLIRT with 12 degrees of freedom, and subsequently refined using non-linear steps as implemented in FSL FNIRT.

***MRI Processing***

Nodes of interest in this study were the 32 cortical and cerebellar regions (or ROIs) from the network atlas as provided by the Conn toolbox (atlas is based on 497 subjects from the Human Connectome Project, see **Table S2**).

**Table S2.** Abbreviation, labels and coordinates of cortical and cerebellar regions that has been used in this study.

| Abbreviation | Conn ROI ID | Left/Right | (X,Y,Z) |
| --- | --- | --- | --- |
| MPFC | DefaultMode.MPFC |  | (1,55,-3) |
| L LP | DefaultMode.LP | (L) | (-39,-77,33) |
| R LP | DefaultMode.LP | (R) | (47,-67,29) |
| PCC | DefaultMode.PCC |  | (1,-61,38) |
| L LSM | SensoriMotor.Lateral | (L) | (-55,-12,29) |
| R LSM | SensoriMotor.Lateral | (R) | (56,-10,29) |
| SSM | SensoriMotor.Superior |  | (0,-31,67) |
| MV | Visual.Medial |  | (2,-79,12) |
| OCC | Visual.Occipital |  | (0,-93,-4) |
| L LV | Visual.Lateral | (L) | (-37,-79,10) |
| R LV | Visual.Lateral | (R) | (38,-72,13) |
| ACC | Salience.ACC |  | (0,22,35) |
| L AI | Salience.AInsula | (L) | (-44,13,1) |
| R AI | Salience.AInsula | (R) | (47,14,0) |
| L RPFC | Salience.RPFC | (L) | (-32,45,27) |
| R RPFC | Salience.RPFC | (R) | (32,46,27) |
| L SMG | Salience.SMG | (L) | (-60,-39,31) |
| R SMG | Salience.SMG | (R) | (62,-35,32) |
| L FEF | DorsalAttention.FEF | (L) | (-27,-9,64) |
| R FEF | DorsalAttention.FEF | (R) | (30,-6,64) |
| IPS | DorsalAttention.IPS | (L) | (-39,-43,52) |
| IPS | DorsalAttention.IPS | (R) | (39,-42,54) |
| L LPFC | FrontoParietal.LPFC | (L) | (-43,33,28) |
| L PPC | FrontoParietal.PPC | (L) | (-46,-58,49) |
| R LPFC | FrontoParietal.LPFC | (R) | (41,38,30) |
| R PPC | FrontoParietal.PPC | (R) | (52,-52,45) |
| L IFG | Language.IFG | (L) | (-51,26,2) |
| R IFG | Language.IFG | (R) | (54,28,1) |
| L pSTG | Language.pSTG | (L) | (-57,-47,15) |
| R pSTG | Language.pSTG | (R) | (59,-42,13) |
| ACB | Cerebellar.Anterior |  | (0,-63,-30) |
| PCB | Cerebellar.Posterior |  | (0,-79,-32) |

**Supplemental Results**

**Association between maternal anxiety in pregnancy and offspring rsFC**

Results of the ANCOVA group comparison, corrected for sex, birth weight (corrected for gestational age), and maternal postnatal anxiety, yielded a significant difference in connectivity between MPFC and left inferior frontal gyrus (IFG), see manuscript. More specifically, this positive correlation was stronger in the LMA offspring (“low-to-medium anxiety”) and weaker in HA offspring (“high anxiety”). A boxplot of the two connectivity distributions for the connection between MPFC and left IFG can be found in **Supplemental Figure S1**.


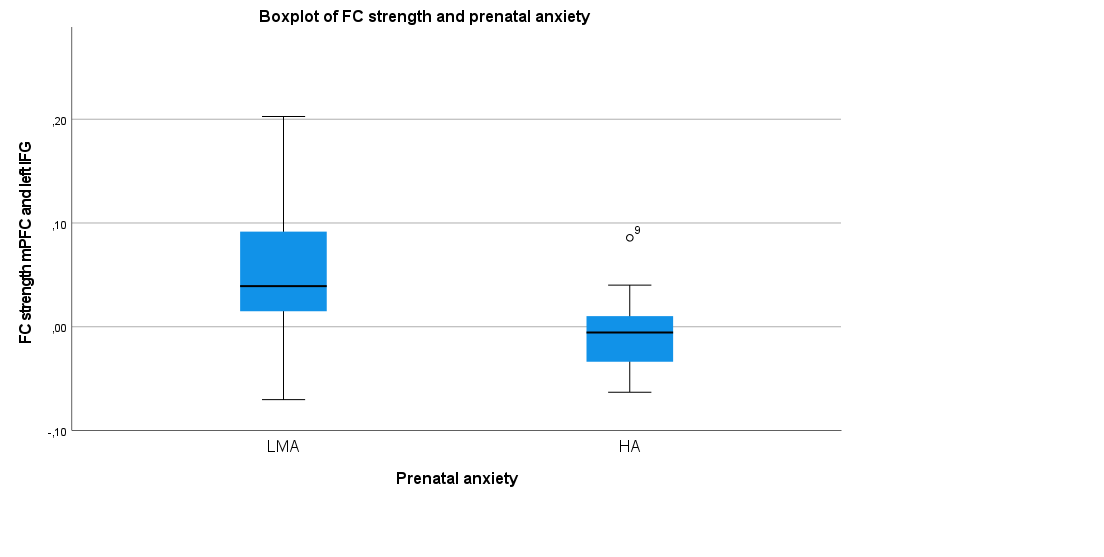


**Figure S1**. **Functional connectivity group-comparison.** Significant group-differences of adult offspring exposed to low-medium maternal anxiety (LMA) and high maternal anxiety (HA) in functional (ROI-to-ROI) connectivity of the medial prefrontal cortex (MPFC) and left inferior frontal gyrus (IFG). Boxplot displays the mean connectivity and 95% intervals for both groups.

**Group differences in graph metrics**

Graph metrics were based on two atlases. Nodes of interest for the first analyses were the 32 cortical and cerebellar regions (or ROIs) from the network atlas as provided by the Conn toolbox (atlas is based on 497 subjects from the Human Connectome Project). The second parcellation was based on the 68 cortical regions of the FreeSurfer’s Desikan Killiany atlas. No group (HA versus LMA) differences were found for global network-based density, connectivity strength, global clustering, normalized global clustering, global efficiency, normalized global efficiency and normalized small worldness (all *p’s*>0.05, see **Table S3**). Results remained non-significant when controlled for covariates sex, birth weight (adapted to gestational age), and maternal postnatal anxiety.

**TableS3**. P-values of differences in graph metrics between Low-Medium Anxiety group and High Anxiety group

|  | **Conn 32 atlas** | **Conn 32 atlas controlled for covariates** | **DK 68 atlas** | **DK 68 atlas controlled for covariates** |
| --- | --- | --- | --- | --- |
| **Graph metrics** |  |  |  |  |
| Density | p=.32 | p=.71 | p=.18 | p=.49 |
| Strength | p=.27 | p=.67 | p=.17 | p=.47 |
| Global clustering | p=.13 | p=.54 | p=.17 | p=.45 |
| Normalized global clustering | p=. 83 | p=.93 | p=.63 | p=.94 |
| Global efficiency | p=.21 | p=.61 | p=.15 | p=.46 |
| Normalized global efficiency | p=.31 | p=.46 | p=.16 | p=.41 |
| Normalized small worldness | p=.79 | p=.66 | p=.95 | p=.83 |

**References**

van Buuren, S., & Groothuis-Oudshoorn, K. (2011). mice: Multivariate Imputation by Chained Equations in R. *Journal of Statistical Software*, *45*(3), 1–67. <https://doi.org/10.18637/jss.v045.i03>

Pruim, R. H. R., Mennes, M., van Rooij, D., Llera, A., Buitelaar, J. K., & Beckmann, C. F. (2015). ICA-AROMA: A robust ICA-based strategy for removing motion artifacts from fMRI data. *Neuroimage*, *112*, 267-277. <https://doi.org/10.1016/j.neuroimage.2015.02.064>

Smith, S. M., Jenkinson, M., Woolrich, M. W., Beckmann, C. F., Behrens, T. E., Johansen-Berg, H., Bannister, P. R., De Luca, M., Drobnjak, I., Flitney, D. E., Niazy, R. K., Saunders, J., Vickers, J., Zhang, Y., De Stefano, N., Brady, J. M., & Matthews, P. M. (2004). Advances in functional and structural MR image analysis and implementation as FSL. *Neuroimage*, *23 Suppl 1*, S208-219. <https://doi.org/10.1016/j.neuroimage.2004.07.051>
